# Cardiac immune cell infiltration associates with abnormal lipid metabolism

**DOI:** 10.3389/fcvm.2022.948332

**Published:** 2022-08-17

**Authors:** Vincenza Cifarelli, Ondrej Kuda, Kui Yang, Xinping Liu, Richard W. Gross, Terri A. Pietka, Gyu Seong Heo, Deborah Sultan, Hannah Luehmann, Josie Lesser, Morgan Ross, Ira J. Goldberg, Robert J. Gropler, Yongjian Liu, Nada A. Abumrad

**Affiliations:** ^1^Department of Medicine, Washington University School of Medicine, St. Louis, MO, United States; ^2^Department of Pharmacology and Physiology, Saint Louis University School of Medicine, St. Louis, MO, United States; ^3^Institute of Physiology, Czech Academy of Sciences, Prague, Czechia; ^4^Division of Complex Drug Analysis, Office of Testing and Research, U.S. Food and Drug Administration, St. Louis, MO, United States; ^5^Department of Radiology, Washington University School of Medicine, St. Louis, MO, United States; ^6^Division of Endocrinology, Department of Medicine, New York University Grossman School of Medicine, New York, NY, United States; ^7^Department of Cell Biology and Physiology, Washington University School of Medicine, St. Louis, MO, United States

**Keywords:** CD36, PET tracers, cardiac inflammation, lipidomics, eicosanoids, macrophage

## Abstract

CD36 mediates the uptake of long-chain fatty acids (FAs), a major energy substrate for the myocardium. Under excessive FA supply, CD36 can cause cardiac lipid accumulation and inflammation while its deletion reduces heart FA uptake and lipid content and increases glucose utilization. As a result, CD36 was proposed as a therapeutic target for obesity-associated heart disease. However, more recent reports have shown that CD36 deficiency suppresses myocardial flexibility in fuel preference between glucose and FAs, impairing tissue energy balance, while CD36 absence in tissue macrophages reduces efferocytosis and myocardial repair after injury. In line with the latter homeostatic functions, we had previously reported that CD36^–/–^ mice have chronic subclinical inflammation. Lipids are important for the maintenance of tissue homeostasis and there is limited information on heart lipid metabolism in CD36 deficiency. Here, we document in the hearts of unchallenged CD36^–/–^ mice abnormalities in the metabolism of triglycerides, plasmalogens, cardiolipins, acylcarnitines, and arachidonic acid, and the altered remodeling of these lipids in response to an overnight fast. The hearts were examined for evidence of inflammation by monitoring the presence of neutrophils and pro-inflammatory monocytes/macrophages using the respective positron emission tomography (PET) tracers, ^64^Cu-AMD3100 and ^68^Ga-DOTA-ECL1i. We detected significant immune cell infiltration in unchallenged CD36^–/–^ hearts as compared with controls and immune infiltration was also observed in hearts of mice with cardiomyocyte-specific CD36 deficiency. Together, the data show that the CD36^–/–^ heart is in a non-homeostatic state that could compromise its stress response. Non-invasive immune cell monitoring in humans with partial or total CD36 deficiency could help evaluate the risk of impaired heart remodeling and disease.

## Introduction

Myocardial energy metabolism is a highly dynamic process important for tissue health and optimal cardiac function. Long-chain fatty acids (FAs) are a major metabolic substrate of myocardial tissue. Rates of tissue FA uptake are dependent on the FA transporter CD36 (1, 2) and its vesicular recycling between endosomes and the sarcolemma (3). CD36 deficiency reduces heart FA uptake and prevents the fasting-induced metabolic remodeling by the myocardium from glucose utilization to more reliance on FAs; following an overnight fast, mice with global Cd36 deletion (CD36^–/–^) displayed atrioventricular block and bradycardia, and increased incidence of sudden death (4). Suppressed or absent myocardial FA uptake has been reported in people with *CD36* gene variants that reduce protein levels (5–7). In addition, relatively common single nucleotide polymorphisms (SNPs) in the *CD36* gene have been associated with cardiac function and with increased susceptibility to cardiovascular disease, as reviewed elsewhere (8). Despite the above findings, how CD36 deficiency impacts heart lipid metabolism and its ability for adaptive remodeling remains unexplored.

The heart is a continuously working organ that adapts to various stresses and its ability to cope and recover is integral to maintaining its health and function. In addition to metabolic remodeling, myocardial recovery relies on the sequential mobilization of immune cells (i.e., neutrophils, monocytes, and macrophages) that serve diverse roles in the reparative process (9, 10), including removal of dead cells and renewal of extracellular matrix (11). Immune cell recruitment in the heart is orchestrated by chemokines interacting with corresponding receptors on leukocytes, mediating their activation and extravasation into the injured area (12–14). Of these chemokine receptors, C-C chemokine receptor type 2 (CCR2), expressed on monocytes and macrophages (15), plays an important role in regulating the phenotype and function of cell types involved in myocardial remodeling, while C-X-C motif chemokine receptor 4 (CXCR4), highly expressed on neutrophils (16), regulates phenotype and function of all cell types involved in tissue healing, making it an important target for both imaging and therapy (17–19). Mice with myeloid CD36 deficiency present defective heart remodeling and repair following injury (20, 21); however, there is no information on whether CD36 deficiency causes immune cell infiltration in unchallenged hearts, as we previously reported in the intestine of CD36^–/–^ mice (22). Such information would be important for evaluating cardiac ability for optimal recovery from injury and potential risk of disease. CD36 deficiency is relatively common in certain populations (3–10%) and *CD36* SNPs that reduce CD36 levels result in dyslipidemia and increase the risk of type 2 diabetes (23, 24).

In this study, we profiled lipid metabolism in the hearts of fed and overnight-fasted mice. Our data identified significant changes in the content of plasmalogens, cardiolipins, and eicosanoids suggesting that CD36-mediated FA delivery is important for maintaining the normal heart lipid profile during the adaptation to fasting. Unbiased global assessment of gene expression by microarray analysis in hearts from CD36^–/–^ mice, as compared to WT, identified upregulation of pathways regulating both innate and adaptive immunity. To gain insight into the dynamic expression and spatial localization of immune cells in unchallenged CD36^–/–^ mice heart, we employed a novel positron emission tomography (PET) imaging approach to non-invasively image neutrophils and pro-inflammatory monocytes/macrophages, using validated tracers ^64^Cu-AMD3100 for CXCR4 and ^68^Ga-DOTA-ECL1i for CCR2 (25, 26). We show that the hearts of unchallenged CD36^–/–^ mice have increased pro-inflammatory immune infiltration as compared to controls. The presence of inflammation in hearts with CD36 deletion specific to cardiomyocytes supported the interpretation that a healthy heart lipid profile is needed to prevent inflammation.

## Materials and methods

### Mice

Mice were bred and maintained at the Washington University School of Medicine and all experimental procedures followed the guidelines of the animal use oversight committee. The studies used cohorts of male and female C57Bl6 wild-type (WT) and CD36-null (CD36^–/–^) mice that were age-matched (12–14 weeks), unless indicated otherwise. Myocardial CD36 deficiency (MHC-CD36^–/–^) was obtained by crossing CD36 floxed (Fl/Fl) mice with mice expressing the myosin heavy chain alpha (MHC) Cre as previously described (27). Mice housed in a 12-h light-dark facility were fed chow *ad libitum* (Purina) or 12 h fasted. Genotypes were confirmed by PCR and immunohistochemistry.

### Lipid analysis

Lipids were extracted and analyzed by mass spectrometry. In brief, hearts from mice killed by carbon dioxide inhalation were rapidly removed, rinsed with ice-cold PBS, freeze-clamped, and pulverized at liquid nitrogen temperature. The tissue was homogenized in LiCl solution (50 mM) using a Potter-Elvehjem tissue grinder. Methanol and chloroform, as well as internal standards for major lipids, were added and the lipids extracted. Multidimensional shotgun lipidomic analysis of extracted lipids used a Thermo Electron TSQ Quantum Ultra spectrometer (San Jose, CA) equipped with an electrospray ion source, and individual molecular species were identified and quantified by 2D mass spectrometry (28–30).

### Metabolite assays

ATP was extracted from hearts (31) and quantified using mass spectrometry (32). Analysis of carnitine and acylcarnitines was performed as described (33). Around 20 mg of heart tissue was lyophilized for 12 h after the addition of a set of deuterium-labeled carnitine and acylcarnitine standards (Cambridge Isotope Laboratories, Andover, MA). The lyophilized tissue was grounded to powder using an Eppendorf micropestle and dissolved in 1 mL of 8:2 (v/v) acetonitrile/water. After sonification and centrifugation, the supernatants were dried and derivatized (33). Carnitine and acylcarnitines were analyzed as their butyl esters by precursor ion scanning of m/z 85 utilizing a TSQ Quantum Ultra Plus triple-quadrupole mass spectrometer (Thermo Fisher Scientific, San Jose, CA). Protein content was measured (Bradford assay, Bio-Rad) with BSA as standard.

### Mitochondria isolation and functional studies

The heart was minced, added to the buffer (10 mM HEPES, 250 mM sucrose, 1mM EGTA, pH 7.0), and homogenized using a Dounce grinder. Heart mitochondria were isolated by differential centrifugation. After centrifugation (500 × g, 2 min) to remove tissue debris, the supernatant was centrifuged at 10,000 xg (10 min) to pellet mitochondria, which were washed once and resuspended at 20 ∼ 30 mg/mL protein at 4°C (0.1 M KCl, 0.05 M Tris-HCl, 2 mM EGTA, pH 7.4). Mitochondrial FA oxidation was measured by monitoring either CO_2_ or water release using [^14^C]-palmitate as substrate. In brief, mitochondria (0.5 mg protein/mL) were incubated with 100 μM FA complexed to BSA (FA:BSA = 1.7) in respiration buffer (120 mM KCl, 5 mM KH2PO4, 3 mM HEPES, 3 mM MgCl2, 1 mM EGTA, 5 mM ATP 1mM NAD, 0.5 mM Carnitine, 0.1 mM Coenzyme A, 5 mM Malate, pH 7.2) aerated with 95% O_2_ and 5% CO_2_. The reaction was terminated by adding hydrochloric acid and radiolabeled products were quantified. Oxygen consumption was measured polarographically using a dual channel Instech Dissolved O_2_ Measuring system. Mitochondria were placed in the electrode chamber in 150 mM KCl, 1 mM EGTA, 5 mM DTT, pH 7.0 at 20°C and the O_2_ consumed was measured for 5 min with no additives and with 5 mM malate added. ADP was added to determine the substrate-supported O_2_ consumption and the ability to generate ATP.

### Gene expression analysis

Gene expression was analyzed by microarray as previously described (4) or by RNA-Seq (27) as indicated. In brief, total RNA was isolated from tissues using Trizol (Invitrogen, Carlsbad, CA). In brief, flash frozen hearts were homogenized in Trizol and chloroform was added (0.2 mL/mL Trizol) followed by centrifugation. The supernatant was removed, an equal volume of isopropanol was added, and the samples were centrifuged again to pellet the RNA, which was washed in 75% ethanol, dried, and resuspended in UltraPure Distilled Water (GIBCO, Carlsbad, CA). Microarray analyses were performed using the Whole Mouse Genome Oligo Microarray Kit (Agilent Technologies, Santa Clara, CA). The array was scanned by Axon 4000B scanner, and the data were extracted by GenePix Pro 6.1 software (Molecular Devices, Sunnyvale, CA). For the RNAseq, raw sequencing data were obtained as previously described in FASTQ format. Read mapping used Tophat 2.0.9 against the mm10 mouse reference genome. The resulting BAM alignment files were processed using HTSeq 0.6.1 python framework and respective mm10 GTF gene annotation (UCSC database). The Bioconductor package DESeq2 (3.2) was used to identify differentially expressed genes (DEGs) and for statistical analysis based on the negative binomial distribution model. The resulting values were adjusted (Benjamini–Hochberg method for FDR determination). Genes with adjusted *P*-value < 0.05 were determined to be differentially expressed. KEGG analysis was used to identify the top canonical pathways being altered. RNA-seq data were deposited in the NCBI’s Gene Expression Omnibus (GEO) database (GEO GSE116350).

### Macrophage polarization and treatments

Bone marrow-derived macrophages were isolated and cultured in RPMI with 10% FBS, 10% L929 conditioned media, and 1% PenStrep for 5 days. For M1 polarization, macrophage media was supplemented with 20 ng/mL IFNγ (Peprotech) and 20 ng/mL lipopolysaccharides (all from Sigma). For M2 polarization, macrophage media was supplemented with 20 ng/mL interleukin-4 (Peprotech). For linoleic acid (LA) and docosahexaenoic acid (DHA) solution: 25 μM LA or DHA (Cayman) complexed with 8 μM FA-Free BSA in PBS supplemented with glucose and 2 μM calcium chloride. Solutions were prepared fresh 24 h before treatment and rotated at 4°C overnight to allow for complete complexing to BSA. Eicosanoids were measured by liquid chromatography–mass spectrometry (LC–MS) as previously described (34).

### Positron emission tomographic/computed tomography imaging

Mice were fasted for 4 h, anesthetized with isoflurane, and injected with 3.7 MBq of ^64^Cu-AMD3100 (^64^Cu: t_1/2_ = 12.7 h) or 9.25 MBq ^68^Ga-DOTA-ECL1i (^68^Ga: t_1/2_ = 68 min) in 100 μL of saline *via* the tail vein. Small animal PET/CT scans (40–60 min dynamic scan) were performed on the Inveon PET/CT system (Siemens, Malvern, PA). The PET images were corrected for attenuation, scatter, normalization, and camera dead time and co-registered with CT images. The PET images were reconstructed with the maximum *a posteriori* (MAP) algorithm and analyzed by Inveon Research Workplace. The uptake was calculated as the percent injected dose per gram (%ID/g) of tissue in three-dimensional regions of interest (ROIs) without the correction for partial volume effect (26).

### Flow cytometry

Single-cell suspensions were generated from saline perfused hearts by finely mincing and digesting them in DMEM with Collagenase 1 (450 U/mL), Hyaluronidase (60 U/mL), and DNase I (60 U/mL) for 1 h at 37°C. All enzymes were purchased from Sigma. To deactivate the enzymes, samples were washed with HBSS that was supplemented with 2% FBS and 0.2% BSA and filtered through 40 μM cell strainers. Red blood cell lysis was performed with ACK lysis buffer (Thermo Fisher Scientific). Samples were washed with HBSS and resuspended in 100 μL of FACS buffer (DPBS with 2% FBS and 2 mM EDTA). Cells were stained with monoclonal antibodies at 4°C for 30 min in the dark. All the antibodies were obtained from Biolegend: CD45-PerCP/Cy5.5, clone 30-F11; CD64-APC and PE, clone X54-5/7.1; CCR2-BV421, clone: SA203G11; MHCII-APC/Cy7, clone M5/114.15.2; Ly6G-PE/Cy7, clone 1A8; and Ly6C-FITC, clone HK1.4. Samples were washed two times, and final resuspension was made in 300 μL FACS buffer. DAPI or LIVE/DEAD™ Aqua dyes were used for the exclusion of dead cells. Flow cytometric analysis were performed on the BD Fortessa platform. Neutrophils were gated as CD45 + Ly6G^high^. Macrophages were gated as Ly6G^neg^CD64^high^Ly6C^low^ cells.

### Statistics

Statistical analyses were made using GraphPad Prism 8 or MATLAB Student’s *t*-test, one-way or two-way ANOVA with *post hoc* comparison. Principal component analysis (PCA) using Umetrics SIMCA-P + 12 software (Umetrics AB) was conducted for non-biased evaluation of lipidomic data (22). All data presented are means ± standard error (SE). Significance was for *p* < 0.05.

## Results

### Cardiac lipidomic profile

**FIGURE 1:**
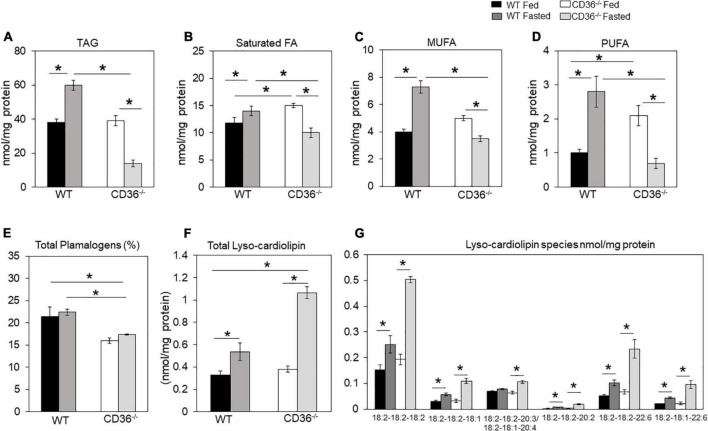
CD36-mediated alterations in myocardial triglycerides and lysocardiolipin. Myocardial lipids in fed and fasted WT and CD36^–/–^ mice were analyzed using shotgun lipidomics mass spectrometry. Levels of **(A)** total triglycerides, **(B)** saturated, **(C)** monounsaturated (MUFA), and **(D)** polyunsaturated (PUFA) FAs in TGs. **(E)** Levels of total plasmalogens (%). **(F)** Levels of lysocardiolipins. **(G)** Lysocardiolipin species. Labels denote the number of carbons: number of double bonds. Results are reported as nmol/mg of protein. Data (*n* = 5/group) are means ± SE with *n* representing the number of mice per group, *p < 0.05.

FAs are structural components of membranes, signaling molecules, and energy sources. In addition, they regulate gene expression by providing substrate for histone acetylation (35, 36). The inability of the myocardium to adapt FA utilization and FA oxidation to FA availability disrupts homeostasis and can associate with stress and inflammation. We examined the lipid profile of the CD36^–/–^ heart by conducting an unbiased global assessment using hearts from fed or overnight fasted CD36^–/–^ and WT mice. Turnover of intramyocardial triglycerides (TAG) contributes an estimated 10% of cardiac energy (37); TAG content was similar in hearts of fed WT and CD36^–/–^ mice (Figure 1A). TAG increased after fasting in WT hearts as previously reported (30, 34), in contrast, CD36^–/–^ hearts showed a marked (∼60%) reduction of TAG stores (Figure 1A). We examined FA composition of the TAG to gain insight into TAG remodeling. Saturated FA (Figure 1B) and PUFA (Figure 1D) were significantly increased in the hearts of CD36^–/–^ mice as compared to controls during the fed state. Fasting is associated in WT hearts with increases in TAG content of monounsaturated (>200%) and polyunsaturated (160%) FAs more than saturated FAs (∼130%). In CD36^–/–^ hearts, fasting reduced all FAs in TAG with a larger drop in monounsaturated (-65%) and polyunsaturated (-62%) FAs as compared to saturated FAs (-45%). These results suggested abnormalities of FA desaturation (Figures 1B–D). In line with the results in the fasting state, expression of the myocardial isoform of stearoyl-CoA desaturase increased three-fold in the hearts of fasted WT mice and was 60% reduced in the hearts of fed or fasted CD36^–/–^ mice (data not shown). Myocardial plasmalogens and cardiolipins (CL), two classes of lipids important for the function of peroxisomes and mitochondria in FA oxidation (38) were altered in CD36^–/–^ mice. Plasmalogen levels were reduced (∼25%) in fed and fasted states, as compared with hearts of WT mice (Figure 1E). Fasting increased lysocardiolipin levels in hearts from both genotypes, although the increase was larger in hearts from CD36^–/–^ as compared to those of WT mice, 1.6-, and 2.8-fold, respectively (Figure 1F). Fasting changed FA composition of cardiolipin acyl chains, which is regulated in concert with the remodeling of other phospholipids. Lysocardiolipin is formed by phospholipase removal of FA-acyl chains and acyltransferase mediates reacylation back to cardiolipin. The larger increase in lysocardiolipin in fasted CD36^–/–^ hearts involved all species with the most affected acyl chains being linoleic acid (C18:2) and DHA (C22:6) (Figure 1G).

**FIGURE 2:**
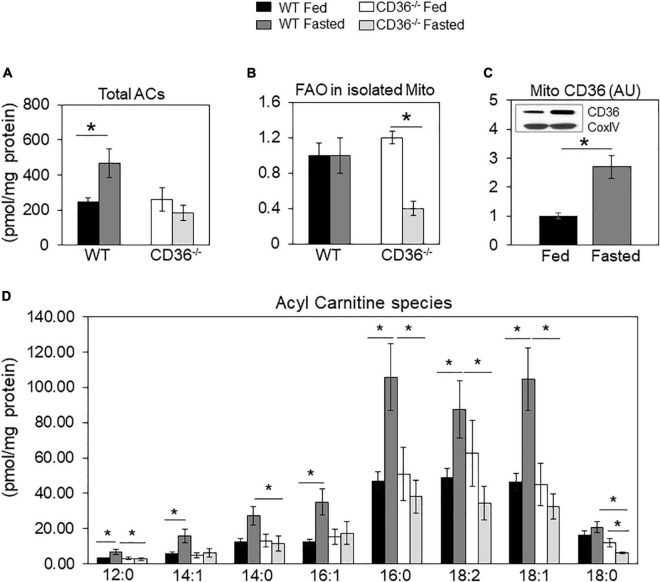
Acylcarnitines and fatty acid oxidation (FAO) of mitochondria isolated from fed and fasted WT and CD36^–/–^ hearts. **(A)** Levels of total acylcarnitines (ACs). **(B)** Mitochondria were isolated to measure CD36 content and fatty acid oxidation (FAO) rate determined from CO_2_ or ^3^H water release from radiolabeled substrates as described in Methods. Data are expressed as ratios of FAO rates from fasted and fed hearts. **(C)** CD36 expression and densitometry in mitochondrial lysates relative to COX IV expression by western blotting. **(A,B)** *n* = 5/group; **(C)** *n* = 7/group; **(D)** *n* = 3/group. Results are reported as pmol/mg of protein. Data are means ± SE with *n* representing the number of mice per group, *p < 0.05.

Tissue renewal relies on lipid metabolism, notably FA oxidation which maintains competent stem cells (39) and regulates cardiac function and homeostasis (8, 40). In the fed state, there was little change in levels of total acylcarnitines (ACs) in CD36^–/–^ hearts (Figure 2A), but while total acylcarnitine levels increased with fasting in WT hearts, they trended lower in CD36^–/–^ hearts, although total AC content is not as meaningful with respect to the status of FA oxidation as that of long-chain ACs. The WT hearts sustained FA oxidation in fasting (Figure 2B) and mitochondrial CD36 protein content increased (Figure 2C) while relative FAO decreased with fasting in CD36^–/–^ hearts (Figure 2B). The heart during fasting normally reduces its glucose utilization and increases its reliance on FA uptake and FA oxidation. However, this metabolic flexibility is lost in the hearts of CD36^–/–^ mice which continue to rely on glucose utilization during fasting (8). In line with this, ACs increased during fasting in the hearts of WT mice, but the increase was uniformly muted in CD36^–/–^ mice as ACs showed little change or decreased (Figure 2D). Levels of acylcarnitines were similar in the fed state in WT and CD36^–/–^ hearts, but during fasting, long-chain AC species (12:0, 16:0, 18:0, 18:1, and 18:2) trended lower but only AC 12:0 and 18:0 reached significance. As compared to the WT heart, long-chain acylcarnitines (12:0, 14:0, 16:0, 18:0, 18:1, and 18:2) were significantly reduced in the hearts of CD36^–/–^ mice indicating reduced mitochondrial FA oxidation. These results are consistent with findings showing that during feeding, the CD36^–/–^ heart can utilize FAs from chylomicrons as uptake of chylomicron FA is not limited by CD36 (8).

**FIGURE 3:**
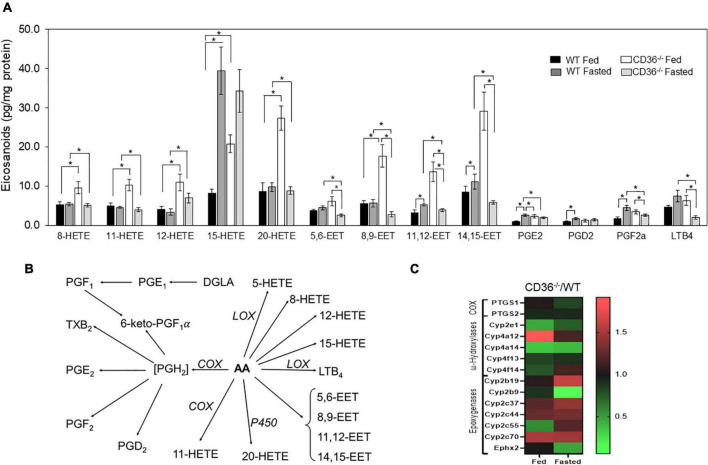
CD36 deficiency affects cardiac eicosanoid content. **(A)** Eicosanoid content in fed or fasted WT and CD36^–/–^ heart (*n* = 5/group). Results are reported as pg/mg of protein. **(B)** Gene expression fold change of key enzymes regulating eicosanoid production. Data are means ± SE with *n* representing the number of mice per group, *p < 0.05. **(C)** The scheme represents downstream metabolites of arachidonic acid (AA) *via* cyclooxygenase (COX), lipoxygenase (LOX), or cytochrome (P450) pathways.

The FAs liberated by phospholipases serve as the primary precursor pool for the formation of the pleiotropically bioactive arachidonic acid-derived eicosanoid generated by cyclooxygenases (COX), lipoxygenases (LOX), and cytochromes P450. The generated eicosanoids are involved in multiple signaling pathways in the heart (41). The role of CD36 in phospholipase A2 activation, release of arachidonic acid, and generation of prostaglandin E2 (PGE2) was previously reported in isolated macrophages (42). We found that levels of many eicosanoids were elevated in the hearts of fed CD36^–/–^ mice as compared to those of WT mice (Figure 3A, black to white bars). This included several LOX-generated hydroxyeicosatetraenoic acids; 8, 12, and 15 HETE and P450 generated epoxyeicosatrienoic acids; 5–6, 8–9, 11–12, and 14–15 EET and 20-HETE. The COX-derived PGE2, PGF2a, and PGD2 were not affected but they increased with fasting only in WT hearts (Figures 3A,B). CD36 deficiency is associated with higher gene expression of the CYP4A arachidonic acid ω-hydroxylases *Cyp4a12* and epoxygenase *Cyp2c70* (Figure 3C). The increase in arachidonic acid-derived eicosanoids during feeding could reflect the presence of inflammation in the myocardium.

**FIGURE 4:**
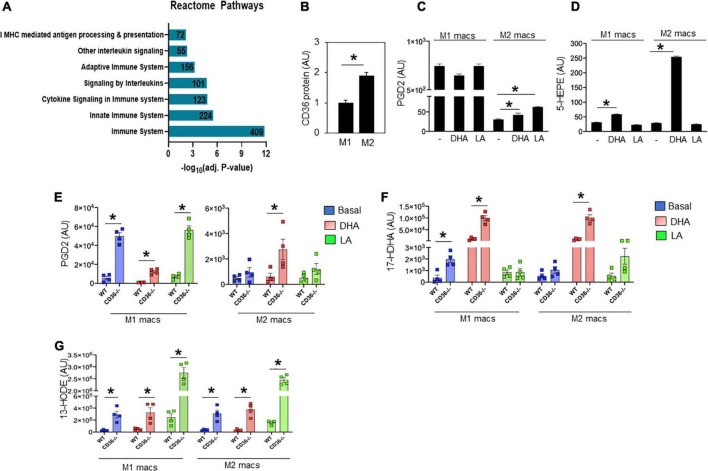
CD36 deletion associates with markers of inflammation in heart tissue and isolated macrophages. **(A)** Microarray analysis in CD36^–/–^ heart shows upregulated biological pathways involved in immune cell activation and inflammation (*n* = 3/group). Pathway analysis obtained from Reactome analysis database using differentially regulated genes (>1.6 or <0.7). Number of differentially regulated genes is indicated on the significance bar for each pathway. **(B)** WT bone marrow-derived macrophages (BMDM) were subjected to a polarization protocol to yield M1- and M2-like macrophages and were assayed for CD36 expression levels by western blotting. WT M1- and M2-like macrophages were assayed for the content of **(C)** PGD2 and **(D)** 5-HEPE at baseline and following DHA or LA treatment. **(E–G)** BMDM were obtained from WT or CD36^–/–^ mice and subjected to the polarization protocol. M1- and M2-like macrophages were then treated with DHA or LA to measure the content of **(E)** PGD2, **(F)** the DHA metabolite 17-Hydroxy-ocosahexaenoic acid (17-HDHA) and **(G)** the linoleic acid metabolite 13-Hydroxyoctadecadienoic acid (13-HODE). Eicosanoids were measured by LC–MS. All data (*n* = 4/group) are means ± SE with *n* representing the number of mice per group. Statistical significance is determined by Student’s *t* test. *p < 0.05.

Unbiased global assessment of gene expression by microarray analysis in hearts of CD36^–/–^ mice, as compared to WT, identified upregulation of pathways regulating both innate and adaptive immunity (Figure 4A and Supplementary Table 1). Excessive or abnormal FA metabolism can promote cardiac inflammation and heart disease which could involve effects on macrophage (MAC) polarization (43). We examined the response of M1-like vs. M2-like MACs to pro-inflammatory (e.g., ω-6 linoleic acid, LA) or anti-inflammatory (e.g., ω-3 docosahexaenoic acid, DHA) polyunsaturated long chain FAs (PUFAs) and the effect of CD36 deficiency. The ω-3 and ω-6 PUFAs are metabolized to various eicosanoids that can, respectively, promote (*via* prostaglandins) or resolve (*via* resolvins) inflammation (44, 45). As reported (46), alternatively polarized M2-like bone marrow-derived MACs have higher CD36 expression when compared to classical M1 MACs (Figure 4B). Untreated WT M1 MACs produced much more PGD2, a metabolite of the ω-6 FA arachidonic acid, than WT untreated M2 MACs (∼843 ± 15 vs. 35 ± 2 AU, respectively, *p* < 0.0001), and M1 PGD2 production was unaffected by FA treatment (Figure 4C). The WT M2 MACs generated low amounts of PGD2 that modestly increased upon addition of DHA (65 ± 4 AU; *p* < 0.05) or LA (80 ± 4 AU; *p* < 0.05) (Figure 4C). We then examined the production of 5-HEPE, a common anti-inflammatory metabolite of ω-3 FAs. Wildtype M1 MACs produced low amounts of 5-HEPE, which modestly increased in response to DHA while a much larger (∼six-fold) increase in 5-HEPE was observed in DHA-treated WT M2 MACs (Figure 4D). Similar data were observed with the anti-inflammatory metabolite 18-HEPE (data not shown). The effect of CD36 deletion on MAC production of eicosanoids was examined next (Figures 4E–G). CD36 deletion increased by ∼nine-fold basal PGD2 levels in M1 MACs (49,862 ± 3,708 AU; *p* < 0.0001) with no further increase observed with LA treatment. DHA suppressed PGD2 levels (12,364 ± 1,377 AU; *p* < 0.001) less efficiently in CD36^–/–^ cells (Figure 4E left panel). DHA treatment increased PGD2 in CD36^–/–^ M2 MACs by ∼ two-fold (to 2,741 ± 811; *p* < 0.05) while LA was ineffective (Figure 4E right panel). These data show that a major effect of CD36 deficiency was to dramatically increase basal PGD2 secretion by M1 MACs. Levels of the anti-inflammatory DHA metabolite 17-HDHA were modestly higher in untreated CD36^–/–^ M1 MACs (1,980 ± 295 AU) as compared to untreated WT M1 MACs (362 ± 242 AU; *P* < 0.01) (Figure 4F). Addition of DHA increased 17-HDHA (11,353 ± 2,530 AU) in WT M1 MACs with a significantly larger effect in CD36^–/–^ cells (99,242 ± 11,230 AU; *p* < 0.001) indicating that CD36 deficiency also increases ω-3 conversion into eicosanoids. A similar pattern was observed in M2 MACs, while LA treatment was generally ineffective (Figure 4F). CD36 deficiency increased (∼10-fold) production of the linoleic acid metabolite 13-HODE in M1 MACs (291,575 ± 57,607 AU) as compared to untreated WT M1 MACs (29,562 ± 5,744 AU; *p* < 0.01), and in response to DHA or LA in both M1 and M2 (Figure 4G). Together, these data suggest that MAC CD36 influences eicosanoid formation, and CD36 loss results in process dysregulation with blunting of differences in eicosanoid production between M1 and M2 MACs.

### Cardiac C-X-C motif chemokine receptor 4 and C-C chemokine receptor type 2 imaging

**FIGURE 5:**
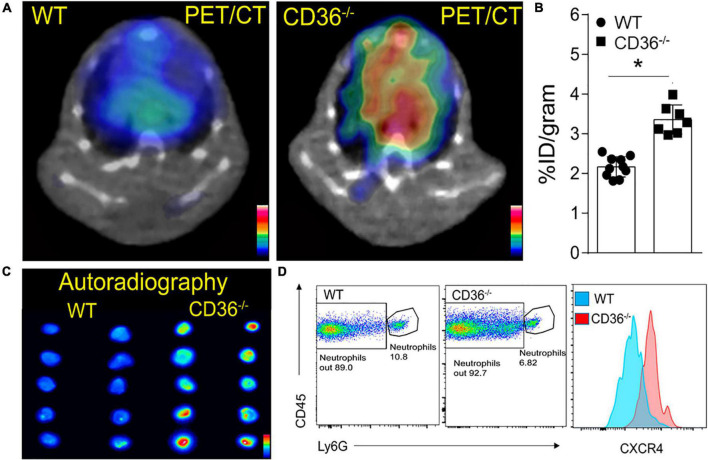
Visualization of activated neutrophils in the heart of wild type (WT) and CD36 *null* (CD36^–/–^) mice. **(A)** Neutrophils are visualized non-invasively using ^64^Cu-AMD3100 tracer and PET/CT scanning. **(B)** Quantification of ^64^Cu-AMD3100 tracer in the heart (*n* = 10 in WT mice, *n* = 7 in CD36^–/–^ mice). **(C)** *Ex vivo* autoradiography of heart slices collected from WT and CD36^–/–^ mice immediately after the PET scan to further document uptake of ^64^Cu-AMD3100 (*n* = 2/group). **(D)** Flow cytometric analysis of CXCR4 expression on cardiac neutrophils isolated from WT and CD36^–/–^ mice (*n* = 3/group). All data are means ± SE with *n* representing the number of mice per group. Statistical significance is determined by the unpaired non-parametric Mann–Whitney *t*-test. *p < 0.05.

**FIGURE 6:**
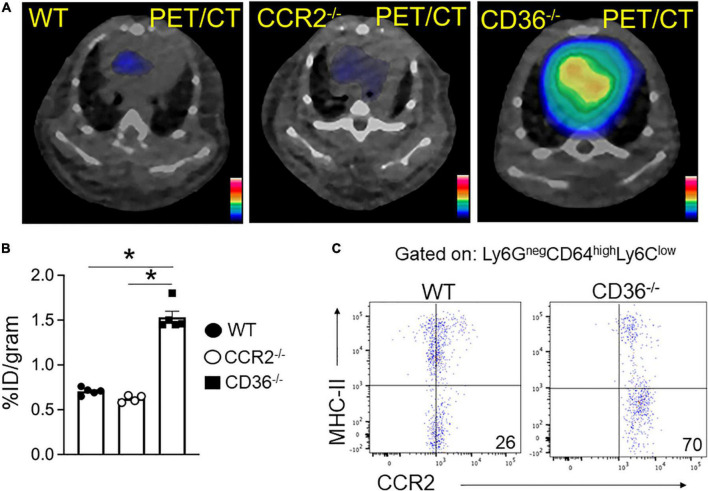
^68^Ga-DOTA-ECL1i PET/CT imaging the infiltration of CCR2 + monocyte/macrophages in hearts. **(A,B)** Representative ^68^Ga-DOTA-ECL1i PET/CT images and quantitative analysis showed higher tracer accumulation in the heart of CD36^–/–^ (*n* = 5) compared with WT mice (*n* = 5) and CCR2^–/–^ mice (*n* = 4/group). **(C)** Flow cytometric analysis of CCR2 expression in macrophages isolated from WT and CD36^–/–^ hearts (*n* = 3/group). All data are means ± SE with *n* representing the number of mice per group. Statistical significance is determined by One-way ANOVA. *p < 0.05.

The above data together suggested the presence of inflammation in the myocardium of the CD36^–/–^ mouse, so we next examined the presence of inflammation non-invasively *in vivo*. Neutrophils are the first cell type to respond when the heart is subjected to stress, infection, or injury. Short-term neutrophil infiltration initiates inflammation and helps with its resolution, while the long-term presence of neutrophils can damage the myocardium (47, 48). High CXCR4 expression is typical of activated neutrophils that can cause tissue damage (49). MAC subsets in the heart play different roles in the tissue’s response to stress. While tissue-resident CCR2^neg^ MACs inhibit monocyte recruitment, CCR2^+^ tissue MACs recruit monocytes and promote cardiac inflammation (50) and contribute to adverse heart remodeling and the pathogenesis of heart failure in humans (51, 52). To non-invasively assess real-time cardiac inflammation in unchallenged CD36^–/–^ mice, we took advantage of a PET-based molecular imaging strategy using ^64^Cu-AMD3100 tracer detecting CXCR4^+^ neutrophils (CXCR4 PET) and ^68^Ga-DOTA-ECL1i tracer detecting CCR2+ macrophages (CCR2 PET) as previously described (25, 26, 53). In CD36^–/–^ mice, robust CXCR4 PET signals were observed in the hearts compared to the low tracer retention measured in wild type controls (Figure 5A). Quantitative analysis showed that tracer uptake in the hearts of CD36^–/–^ mice was significantly higher (3.36 ± 0.36%ID/g, *p* < 0.001, *n* = 7) than that in WT mice (2.17 ± 0.25%ID/g, *n* = 10) (Figure 5B). Moreover, *ex vivo* autoradiography performed immediately after PET/CT imaging showed stronger tracer uptake in heart slices from CD36^–/–^ mice relative to slices from WT mice (Figure 5C). To further validate the CXCR4 PET findings, we determined level of CXCR4^+^ neutrophils in mouse hearts using flow cytometry. CD36^–/–^ hearts as compared to WT hearts, had more CXCR4^+^ neutrophils but not more total neutrophils (CD45^+^Ly6G^+^) (Figure 5D). For CCR2 imaging, higher tracer uptake was measured in CD36^–/–^ mice that contrasted with the low retention of ^68^Ga-DOTA-ECL1i seen in WT mice (Figures 6A,B). Heart uptake quantification revealed nearly doubled signals in CD36^–/–^ mice (1.33 ± 0.16%ID/g, *n* = 5, *p* < 0.001) relative to those in WT mice (0.72 ± 0.06%ID/g, *n* = 5). Moreover, in CCR2^–/–^ mice, minimal ^68^Ga-DOTA-ECL1i accumulation was observed (0.59 ± 0.04%ID/g, *n* = 4, *p* < 0.001), validating CCR2-targeting specificity of ^68^Ga-DOTA-ECL1i (Figures 6A,B). Flow cytometry analysis confirmed increased CCR2 expression on heart MACs isolated from CD36^–/–^ mice (Figure 6C).

### CD36 deletion in cardiomyocyte associates with cardiac inflammation

**FIGURE 7:**
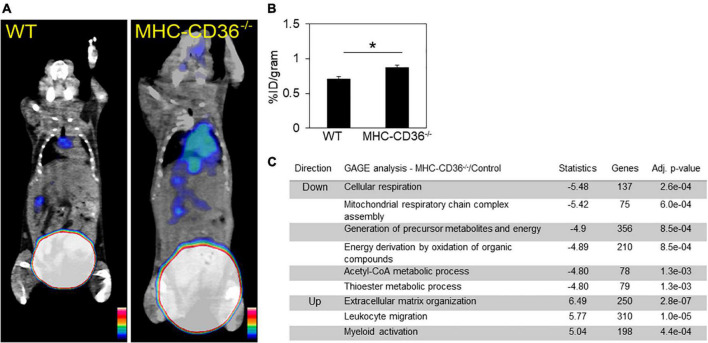
Myocardial CD36 deletion associates with cardiac inflammation and impaired FA metabolisms. **(A,B)** Representative ^68^Ga-DOTA-ECL1i PET/CT images and quantitative analysis showed higher tracer accumulation in the heart of MHC-CD36^–/–^ (*n* = 4/group) compared with those isolated from control mice (*n* = 5/group). Data are means ± SE with *n* representing the number of mice per group, *p < 0.05. **(C)** Gene expression of MHC-CD36^–/–^ and control hearts obtained by RNA-seq. Up- and downregulated biological pathways from Reactome analysis using differentially regulated genes (MHC-CD36^–/–^ vs. WT, > 1.6 or < 0.7-fold change). All data are (*n* = 3/group) with *n* representing the number of mice per group. Number of genes on the differentially regulated list is indicated on significance bar for each pathway.

The alterations in lipid metabolism measured in the hearts of CD36^–/–^ mice would be predicted to cause significant stress to the myocardium, which could promote inflammation. To assess the role of cardiomyocyte CD36 deficiency on cardiac inflammation independent of the context of CD36 deficiency in immune cells, we examined whether inflammation can be observed in hearts from mice with CD36 deletion restricted to cardiomyocytes, MHC-CD36^–/–^ (27). Hearts of MHC-CD36^–/–^ mice had significant increases in the macrophage CCR2 PET tracer as compared to hearts of WT mice (Figures 7A,B). In addition, an unbiased global assessment of gene expression in hearts from MHC-CD36^–/–^ mice showed upregulation of pathways involved in immune cell activation and infiltration. In contrast, pathways relevant to cellular respiration, mitochondrial function, and FA metabolism were downregulated as compared to littermate controls (Figure 7C). These data suggested that CD36 deficiency in cardiomyocytes likely contributes to tissue inflammation and immune infiltration.

## Discussion

Our study examined metabolic and immune adaptation of the heart in the context of CD36-mediated impaired lipid metabolism. Lipidomic analysis in hearts from CD36^–/–^ mice showed abnormal remodeling of TAG and mitochondrial cardiolipin in fasting, and reduced levels of peroxisomal plasmalogens, a major glycerophospholipid class in the myocardium (54). In line with this, fasting associated with suppressed mitochondrial FA oxidation and with reduced FA acylcarnitine levels. Eicosanoid production was dysregulated in fed CD36^–/–^ mice with high levels of multiple pro-inflammatory arachidonic acid derivatives. Analysis of heart gene expression showed that CD36 deficiency promotes the downregulation of genes involved in cellular respiration and mitochondrial function while upregulating the expression of genes involved in immune cell activation and inflammation. Using a novel non-invasive PET imaging approach, we further showed that CD36 deficiency promotes cardiac immune cell infiltration in unchallenged mice. Increases were observed in PET signals for CXCR4, a marker of old activated neutrophils that cause tissue damage (49), and for CCR2 which marks a pro-inflammatory subset of monocytes-macrophages, that associates with adverse left ventricle remodeling and heart failure progression (26, 55). CD36 regulates macrophage polarization to the anti-inflammatory phenotype and improves macrophage efferocytosis after ischemic injury (56). Deficiency in myeloid-CD36 delays the clearing of apoptotic cells, including neutrophils and cardiac repair after injury (21). Uncleared apoptotic neutrophils are implicated in tissue injury through the generation of pro-inflammatory cytokines, extracellular traps, and reactive oxygen species (57). Although neutrophils were recently shown to have a protective role in polarizing macrophages toward a healing function, the absence of CD36 on macrophages would at least in part impede this effect (58, 59). The observation that CD36 deficiency restricted to cardiomyocytes promotes macrophage infiltration and upregulation of pathways related to immune activation and migration in the heart suggests that dysregulation of lipid metabolism might drive the immune infiltration in CD36^–/–^ hearts.

CD36 deficiency in rodents was reported to associate with intolerance to prolonged fasting with increases in arrhythmias and sudden death (4). One contributor to fasting intolerance of CD36^–/–^ mice is likely the fasting-induced TAG depletion. The fasted CD36^–/–^ heart relies more heavily than the WT heart on glucose and endogenous lipid stores and is unable to replenish them by FA supply during fasting. However, in the fed state, the CD36^–/–^ heart can derive FAs from chylomicrons. The heart utilizes FFAs bound to albumin, as well as FFAs, released from very low-density lipoproteins (VLDL) or from intestinally derived chylomicrons *via* vascular lipoprotein lipase (LpL)-mediated enzymatic cleavage of triacylglycerol (TG) ester bonds. A comparison of myocardial FA uptake from VLDL and chylomicrons in mice showed that CD36 was important for FA uptake from VLDL but not from chylomicrons, as the effect of CD36 deletion on FA uptake from chylomicrons was negligible (60). Therefore, in the fed state, the CD36^–/–^ heart can replenish endogenous lipids from chylomicrons. In the fasting state, CD36^–/–^ hearts fail to replenish TAG stores as uptake of FA and VLDL is CD36 dependent (8, 60). Targeting cardiac FA uptake can provide a primary approach for preventing excess lipid accumulation and lipotoxicity as proposed (61) but our findings indicate that very low FA uptake rates can negatively impact myocardial adaptation and the remodeling required for the maintenance of tissue health. In line with the current results with CD36^–/–^ hearts, mice with LpL deficiency restricted to cardiomyocytes showed altered cardiac expression of genes involved in lipid and glucose metabolism (62) and developed heart dysfunction with aging and increased afterload (63).

### Altered remodeling of cardiolipins and plasmalogens

We previously reported that CD36^–/–^ hearts had higher contents of lysophospholipids, LPC, and LPE (4). These earlier findings together with the current observations of abnormal increases in lysocardiolipins are consistent with the interpretation that FA acyl supply might be rate-limiting for phospholipid remodeling in fasted CD36^–/–^ hearts in line with the reduction in FA acyl sources, namely myocardial TAG and FA uptake. However, alternative or additional mechanisms cannot be ruled out. Phospholipid remodeling is regulated by signal transduction events, including changes in calcium dynamics, which are altered in CD36^–/–^ hearts (4). Dysfunction of calcium flux, or activation of phospholipases A2, or lysophospholipid acyltransferases could result in abnormal tissue remodeling. We observed 50% suppression of gene expression for heart cytoplasmic cPLA2 and iPLA2γ, enzymes that mobilize arachidonic from PC and PE to yield lysoPC and lysoPE, and a two to fourfold increase in expression of the acyltransferases LPCAT2 and LPCAT3 that reacylate lysoPC to PC (data not shown). It remains to be determined whether these changes are compensatory in the context of dysregulated enzymatic activity.

Remodeling of cardiolipin side-chains is important for mitochondrial function (64, 65). Linoleic, oleic, arachidonic, and docosahexaenoic acids are major side-chains of mouse heart cardiolipin (66) and are maintained preferentially through acyl chain transfer from the *sn*-2 position of PC (67). Maintaining appropriate levels of the key C18:2 and C22:6 cardiolipin might be suboptimal in CD36^–/–^ hearts. Remodeling of phospholipids is important for myocardial adaptation to stress and is thought to boost sarcoplasmic reticulum Ca^2+^ uptake. For example, altered DHA content of heart phospholipids in mice with deficiency of the very-long-chain acyl-CoA dehydrogenase that initiates oxidation of FA with 14–20 carbons is associated with abnormal Ca^2+^ handling, tachycardia, and prolonged QT interval (68, 69), similar to dysfunctions noted in fasted CD36^–/–^ mice (4). The dysregulated phospholipid (PC, PE, and CL) remodeling induced by CD36 ablation appears maladaptive and might contribute to impairing calcium handling.

Level of plasmalogens was reduced in fed and fasted CD36^–/–^ hearts as compared to controls. Plasmalogens are enriched in plasma membrane lipid-raft microdomains (70) and function as potent endogenous antioxidants regulating susceptibility to oxidative damage in many diseases (71–73). In addition, they are important for mitochondrial FA oxidation (38). Plasmalogens can also generate toxic lysolipids and hydroxy fatty aldehydes and are the target of activated neutrophils (74), but whether the reduction in plasmalogen levels is in part linked to neutrophil infiltration is unclear.

### Changes in eicosanoids

The altered phospholipid remodeling in CD36^–/–^ hearts is associated with increased production of various eicosanoids especially in the fed state when the supply of FAs is abundant. The data suggest that eicosanoid production is dysregulated and that it is normally suppressed by CD36 presence. This is in line with the findings in macrophages where CD36 deficiency upregulated basal or FA-induced eicosanoid production (Figures 4E–G). In macrophages, CD36 deficiency markedly increased levels of the pro-inflammatory PGD2 in M1 macrophages and those of the linoleic acid metabolite 13-HODE by both M1 and M2 macrophages. These lipid mediators influence diverse signaling pathways in the heart including activation of ion channels, modulation of calcium flux, and their resultant effects on cardiac hypertrophy, myocardial preconditioning, infarction, and arrhythmogenesis (41).

CD36^–/–^ hearts have higher levels of most arachidonic-derived eicosanoids as compared to WT hearts. Arachidonic acid metabolism generates mostly pro-inflammatory mediators but can also yield metabolites that help resolve inflammation (75). Arachidonic acid metabolism to prostaglandins *via* the COX pathway or to leukotrienes *via* the LOX pathway is associated with acute inflammation (75). In addition to the increased production of pro-inflammatory eicosanoids, we observed a fourfold increase in cytochrome P450-derived epoxy cardioprotective compounds (76). The CYP pathway produces the pro-resolution mediator epoxyeicosatrienoic acid, thought to increase the recruitment of dendritic cells and monocytes important for repair (75). The EETs, present in the heart, endothelium, and plasma have vasodilatory, anti-inflammatory, antioxidative, anti-migratory, and pro-fibrinolytic effects in the heart, with 11,12-EET being the most efficacious. Some of these anti-inflammatory effects might be mediated by the inhibitory role of EETs on pro-inflammatory mediators in the vascular wall, ICAM-1, VCAM-1, and E-selectin. The 11,12-EET also inhibits phosphorylation of IκB-α, which is necessary for nuclear translocation of NF-κB, preventing activation of NF-κB target genes (77). However, it is worth noting that the changes in EET levels in CD36^–/–^ hearts occurred in opposite direction to those in WT hearts. Levels were much higher in the fed state and declined after fasting to levels below those of WT hearts (e.g., 11,12-EET, and 14,15-EET). Overall, eicosanoid metabolism appears dysregulated in CD36^–/–^ hearts, and further studies are needed to understand the molecular mechanisms driving the changes.

### Is CD36 good or bad for heart homeostasis?

The current findings and our previous observations support the importance of CD36 for maintaining heart metabolic flexibility and optimal adaptation to energy fluctuations. They also suggest an important role of the protein in the regulation of phospholipid remodeling important for membrane function. Emerging research show that CD36 has dual actions in the inflammatory process, as the receptor is reported to regulate both induction and resolution of inflammation possibly due to its capability to bind different ligands. For example, CD36-mediated uptake of oxLDL (78) contributes to cholesterol accumulation in arterial wall macrophages (79). CD36 induces inflammation by forming a complex with TLR4 (80) was shown to regulate nucleation and accumulation of cholesterol crystals within macrophages resulting in activation of the NLRP3 inflammasome (75). On the other hand, as a pattern recognition receptor, CD36 is involved in the clearance of cell debris and phagocytosis, which is important for resolving inflammation by contributing to the clearance of apoptotic neutrophils (81). In the heart, CD36 may be important for the resolution of cardiac inflammation after injury by contributing to monocyte clearance of apoptotic cells (21, 82). Impaired resolution of inflammation is also observed with CD36 deletion in lymphatic endothelial cells and the resulting impairment of the lymphatic barrier (83). These dual actions of CD36 in helping mount and resolve inflammation would be consistent with the concept that normally inflammation is tightly controlled, self-limited, and programs its own resolution (84). In summary, the heart of unchallenged CD36^–/–^ mice is in a non-homeostatic state and displays strikingly abnormal remodeling of tissue lipids. This includes lipids important for energy production, TAG, and cardiolipin, which are associated with diminished mitochondrial oxidation and the reduced production of acylcarnitines during fasting. Abnormal remodeling of membrane phospholipids, important for tissue adaptation to stress, associated with dysregulated production of the bioactive eicosanoids, which play important roles in multiple physiological pathways, including the initiation and resolution of inflammation.

### Potential relevance to humans

CD36 genetic variants that reduce protein level by ∼50% are relatively common, with a minor allele incidence of ∼20% in African Americans. Partial or total CD36 deficiency is associated with abnormal blood lipid profile (24, 85) and with vascular stiffening (23), which are predictors of obesity/diabetes-associated cardiovascular events. Humans deficient in CD36 have impaired FA uptake as visualized by spectral imaging using the FA analog BMIPP. Myocardial BMIPP uptake is almost undetectable in CD36 deficient humans and is significantly reduced in subjects with partial CD36 deficiency (6, 86) suggesting a quantitative gene-dosage dependency. The dysregulated eicosanoid secretion in the hearts of CD36^–/–^ mice might signal the presence of subclinical tissue damage in line with the observed immune infiltration. High levels of eicosanoids have been associated with myocardial vulnerability. Increases in LOX-generated AA-derived HETEs, notably 12-HETE and 15-HETE, and of P450-generated AA-derived 11,12-EET and 14,15-EET were shown to correlate with the N-terminal pro-B-type natriuretic peptide, a cardiac biomarker predictive of the acute coronary syndrome and heart failure. They are also associated with the subsequent onset of acute myocardial infarct in patients with coronary artery disease (87). Our findings warrant further investigation of interventions that would mitigate the negative effects of the dysregulated lipid metabolism in CD36 deficient hearts. It would be important to determine whether polymorphisms that significantly reduce CD36 levels might be predictive of myocardial vulnerability to stress and could be a useful biomarker of individual susceptibility to (i) immune cell-induced cardiac pathological remodeling and (ii) electrical instability during fasting or other catabolic states.

## Data availability statement

RNA-seq data were deposited in the NCBI’s Gene Expression Omnibus (GEO) database (GEO GSE116350).

## Ethics statement

This animal study was reviewed and approved by the Washington University Animal Studies Committee.

## Author contributions

VC, OK, RWG, YL, RG, and NAA designed the research studies and analyzed the data. VC, OK, GSH, DS, HL, JL, KY, MR, TP, IJG, and YL contributed to data collection and analysis. VC, OK, and NAA wrote the manuscript. All authors reviewed the manuscript.

## Abbreviations

PET: Positron emission tomography
CT: Computed Tomography
CXCR4: CXCR4
CCR2: C-C chemokine receptor type 2
DHA: Docosahexaneoic Acid
LA: Linoleic acid
LPC: Lysophosphatidylcholine
LPE: Lysophosphatidylethanolamine
LC: Lysocardiolipins
AC: Acylcarnitine
5-HEPE: Hydroxyeicosapentaenoic acid
17-HDHA: 17-Hydroxy-docosahexaenoic acid
13-HODE: 13-Hydroxyoctadecadienoic acid
PGD2: Prostaglandin D2
PGF1: Prostaglandin F1
PGE: Prostaglandin E
DGLA: Dihomo-γ-linolenic acid
TXB2: Thromboxane B2
LTB4: Leukotriene B4
HETEs: Hydroxyeicosatetraenoic acids
EETs: Epoxyeicosatrienoic acids.

## References

[1] Hames KC Vella A Kemp BJ Jensen MD. Free fatty acid uptake in humans with CD36 deficiency. *Diabetes.* (2014) 63:3606–14. 24917573 10.2337/db14-0369 PMC4207394

[2] Carley AN Bi J Wang X Banke NH Dyck JR O’Donnell JM Multiphasic triacylglycerol dynamics in the intact heart during acute in vivo overexpression of CD36. *J Lipid Res.* (2013) 54:97–106. 10.1194/jlr.M029991 23099442 PMC3520544

[3] Glatz JFC Luiken J Nabben M. CD36 (SR-B2) as a target to treat lipid overload-induced cardiac dysfunction. *J Lipid Atheroscler.* (2020) 9:66–78. 10.12997/jla.2020.9.1.66 32821722 PMC7379071

[4] Pietka TA Sulkin MS Kuda O Wang W Zhou D Yamada KA CD36 protein influences myocardial Ca2+ homeostasis and phospholipid metabolism: conduction anomalies in CD36-deficient mice during fasting. *J Biol Chem.* (2012) 287:38901–12. 10.1074/jbc.M112.413609 23019328 PMC3493931

[5] Okamoto F Tanaka T Sohmiya K Kawamura K. CD36 abnormality and impaired myocardial long-chain fatty acid uptake in patients with hypertrophic cardiomyopathy. *Jpn Circ J.* (1998) 62:499–504. 10.1253/jcj.62.499 9707006

[6] Tanaka T Nakata T Oka T Ogawa T Okamoto F Kusaka Y Defect in human myocardial long-chain fatty acid uptake is caused by FAT/CD36 mutations. *J Lipid Res.* (2001) 42:751–9. 11352982

[7] Tanaka T Sohmiya K Kawamura K. Is CD36 deficiency an etiology of hereditary hypertrophic cardiomyopathy? *J Mol Cell Cardiol.* (1997) 29:121–7. 9040027 10.1006/jmcc.1996.0257

[8] Abumrad NA Goldberg IJ. CD36 actions in the heart: lipids, calcium, inflammation, repair and more? *Biochim Biophys Acta.* (2016) 1861:1442–9. 10.1016/j.bbalip.2016.03.015 27004753 PMC4983248

[9] Forte E Furtado MB Rosenthal N. The interstitium in cardiac repair: role of the immune-stromal cell interplay. *Nat Rev Cardiol.* (2018) 15:601–16. 10.1038/s41569-018-0077-x 30181596

[10] Gentek R Hoeffel G. The innate immune response in myocardial infarction repair, and regeneration. *Adv Exp Med Biol.* (2017) 1003:251–72. 28667562 10.1007/978-3-319-57613-8_12

[11] Weinberger T Schulz C. Myocardial infarction: a critical role of macrophages in cardiac remodeling. *Front Physiol.* (2015) 6:107. 10.3389/fphys.2015.00107 25904868 PMC4387471

[12] Shinagawa H Frantz S. Cellular immunity and cardiac remodeling after myocardial infarction: role of neutrophils, monocytes, and macrophages. *Curr Heart Fail Rep.* (2015) 12:247–54. 25721354 10.1007/s11897-015-0255-7

[13] Swirski FK. Inflammation and repair in the ischaemic myocardium. *Hamostaseologie.* (2015) 35:34–6. 25375277 10.5482/HAMO-14-09-0045

[14] Cavalera M Frangogiannis NG. Targeting the chemokines in cardiac repair. *Curr Pharm Des.* (2014) 20:1971–9. 23844733 10.2174/13816128113199990449 PMC4408530

[15] Geissmann F Jung S Littman DR. Blood monocytes consist of two principal subsets with distinct migratory properties. *Immunity.* (2003) 19:71–82. 10.1016/s1074-7613(03)00174-2 12871640

[16] Nahrendorf M Swirski FK. PET Imaging of Leukocytes in Patients With Acute Myocardial Infarction. *JACC Cardiovasc Imaging.* (2015) 8:1427–9. 26699111 10.1016/j.jcmg.2015.10.004

[17] Frangogiannis NG. The stromal cell-derived factor-1/CXCR4 axis in cardiac injury and repair. *J Am Coll Cardiol.* (2011) 58:2424–6. 22115650 10.1016/j.jacc.2011.08.031

[18] Nishimura Y Ii M Qin G Hamada H Asai J Takenaka H CXCR4 antagonist AMD3100 accelerates impaired wound healing in diabetic mice. *J Invest Dermatol.* (2012) 132(3 Pt 1):711–20. 22048734 10.1038/jid.2011.356 PMC3276738

[19] Jujo K Hamada H Iwakura A Thorne T Sekiguchi H Clarke T CXCR4 blockade augments bone marrow progenitor cell recruitment to the neovasculature and reduces mortality after myocardial infarction. *Proc Natl Acad Sci USA.* (2010) 107:11008–13. 10.1073/pnas.0914248107 20534467 PMC2890743

[20] Irie H Krukenkamp IB Brinkmann JF Gaudette GR Saltman AE Jou W Myocardial recovery from ischemia is impaired in CD36-null mice and restored by myocyte CD36 expression or medium-chain fatty acids. *Proc Natl Acad Sci USA.* (2003) 100:6819–24. 12746501 10.1073/pnas.1132094100 PMC164530

[21] Dehn S Thorp EB. Myeloid receptor CD36 is required for early phagocytosis of myocardial infarcts and induction of Nr4a1-dependent mechanisms of cardiac repair. *FASEB J.* (2018) 32:254–64. 10.1096/fj.201700450R 28860151 PMC5731133

[22] Cifarelli V Ivanov S Xie Y Son NH Saunders BT Pietka TA CD36 deficiency impairs the small intestinal barrier and induces subclinical inflammation in mice. *Cell Mol Gastroenterol Hepatol.* (2017) 3:82–98. 10.1016/j.jcmgh.2016.09.001 28066800 PMC5217470

[23] Shibao CA Celedonio JE Ramirez CE Love-Gregory L Arnold AC Choi L A common CD36 variant influences endothelial function and response to treatment with phosphodiesterase 5 inhibition. *J Clin Endocrinol Metab.* (2016) 101:2751–8. 10.1210/jc.2016-1294 27144937 PMC4929841

[24] Love-Gregory L Kraja AT Allum F Aslibekyan S Hedman AK Duan Y Higher chylomicron remnants and LDL particle numbers associate with CD36 SNPs and DNA methylation sites that reduce CD36. *J Lipid Res.* (2016) 57:2176–84. 10.1194/jlr.P065250 27729386 PMC5321222

[25] Zhao Y Detering L Sultan D Cooper ML You M Cho S Gold nanoclusters doped with (64)Cu for CXCR4 positron emission tomography imaging of breast cancer and metastasis. *ACS Nano.* (2016) 10:5959–70. 10.1021/acsnano.6b01326 27159079 PMC5479491

[26] Heo GS Kopecky B Sultan D Ou M Feng G Bajpai G Molecular imaging visualizes recruitment of inflammatory monocytes and macrophages to the injured heart. *Circ Res.* (2019) 124:881–90. 10.1161/CIRCRESAHA.118.314030 30661445 PMC6435034

[27] Son NH Basu D Samovski D Pietka TA Peche VS Willecke F Endothelial cell CD36 optimizes tissue fatty acid uptake. *J Clin Invest.* (2018) 128:4329–42. 10.1172/JCI99315 30047927 PMC6159965

[28] Han X Yang K Gross RW. Multi-dimensional mass spectrometry-based shotgun lipidomics and novel strategies for lipidomic analyses. *Mass Spectrom Rev.* (2012) 31:134–78. 10.1002/mas.20342 21755525 PMC3259006

[29] Liu X Moon SH Mancuso DJ Jenkins CM Guan S Sims HF Oxidized fatty acid analysis by charge-switch derivatization, selected reaction monitoring, and accurate mass quantitation. *Anal Biochem.* (2013) 442:40–50. 10.1016/j.ab.2013.06.014 23850559 PMC3823533

[30] Han X Yang J Cheng H Ye H Gross RW. Toward fingerprinting cellular lipidomes directly from biological samples by two-dimensional electrospray ionization mass spectrometry. *Anal Biochem.* (2004) 330:317–31. 10.1016/j.ab.2004.04.004 15203339

[31] Ford DA Han X Horner CC Gross RW. Accumulation of unsaturated acylcarnitine molecular species during acute myocardial ischemia: metabolic compartmentalization of products of fatty acyl chain elongation in the acylcarnitine pool. *Biochemistry.* (1996) 35:7903–9. 10.1021/bi960552n 8672492

[32] Su X Han X Mancuso DJ Abendschein DR Gross RW. Accumulation of long-chain acylcarnitine and 3-hydroxy acylcarnitine molecular species in diabetic myocardium: identification of alterations in mitochondrial fatty acid processing in diabetic myocardium by shotgun lipidomics. *Biochemistry.* (2005) 44:5234–45. 10.1021/bi047773a 15794660

[33] Spiekerkoetter U Tokunaga C Wendel U Mayatepek E Ijlst L Vaz FM Tissue carnitine homeostasis in very-long-chain acyl-CoA dehydrogenase-deficient mice. *Pediatr Res.* (2005) 57:760–4. 15774826 10.1203/01.PDR.0000157915.26049.47

[34] Kuda O Rombaldova M Janovska P Flachs P Kopecky J. Cell type-specific modulation of lipid mediator’s formation in murine adipose tissue by omega-3 fatty acids. *Biochem Biophys Res Commun.* (2016) 469:731–6. 10.1016/j.bbrc.2015.12.055 26707880

[35] McDonnell E Crown SB Fox DB Kitir B Ilkayeva OR Olsen CA Lipids reprogram metabolism to become a major carbon source for histone acetylation. *Cell Rep.* (2016) 17:1463–72. 10.1016/j.celrep.2016.10.012 27806287 PMC5123807

[36] Clemot M Senos Demarco R Jones DL. Lipid mediated regulation of adult stem cell behavior. *Front Cell Dev Biol.* (2020) 8:115. 10.3389/fcell.2020.00115 32185173 PMC7058546

[37] Saddik M Lopaschuk GD. Triacylglycerol turnover in isolated working hearts of acutely diabetic rats. *Can J Physiol Pharmacol.* (1994) 72:1110–9. 10.1139/y94-157 7882174

[38] Park H He A Tan M Johnson JM Dean JM Pietka TA Peroxisome-derived lipids regulate adipose thermogenesis by mediating cold-induced mitochondrial fission. *J Clin Invest.* (2019) 129:694–711. 10.1172/JCI120606 30511960 PMC6355224

[39] Ito K Carracedo A Weiss D Arai F Ala U Avigan DE A PML-PPAR-delta pathway for fatty acid oxidation regulates hematopoietic stem cell maintenance. *Nat Med.* (2012) 18:1350–8. 10.1038/nm.2882 22902876 PMC3566224

[40] Shu H Peng Y Hang W Nie J Zhou N Wang DW. The role of CD36 in cardiovascular disease. *Cardiovasc Res.* (2022) 118:115–29. 33210138 10.1093/cvr/cvaa319 PMC8752351

[41] Jenkins CM Cedars A Gross RW. Eicosanoid signalling pathways in the heart. *Cardiovasc Res.* (2009) 82:240–9. 19074824 10.1093/cvr/cvn346 PMC2675928

[42] Kuda O Jenkins CM Skinner JR Moon SH Su X Gross RW CD36 protein is involved in store-operated calcium flux, phospholipase A2 activation, and production of prostaglandin E2. *J Biol Chem.* (2011) 286:17785–95. 10.1074/jbc.M111.232975 21454644 PMC3093854

[43] Mouton AJ Li X Hall ME Hall JE. Obesity, hypertension, and cardiac dysfunction: novel roles of immunometabolism in macrophage activation and inflammation. *Circ Res.* (2020) 126:789–806. 10.1161/CIRCRESAHA.119.312321 32163341 PMC7255054

[44] Werz O Gerstmeier J Libreros S De la Rosa X Werner M Norris PC Human macrophages differentially produce specific resolvin or leukotriene signals that depend on bacterial pathogenicity. *Nat Commun.* (2018) 9:59. 10.1038/s41467-017-02538-5 29302056 PMC5754355

[45] Panigrahy D Gilligan MM Serhan CN Kashfi K. Resolution of inflammation: an organizing principle in biology and medicine. *Pharmacol Ther.* (2021) 227:107879. 10.1016/j.pharmthera.2021.107879 33915177

[46] Orecchioni M Ghosheh Y Pramod AB Ley K. Macrophage polarization: different gene signatures in M1(LPS+) vs. classically and M2(LPS-) vs. alternatively activated macrophages. *Front Immunol.* (2019) 10:1084. 10.3389/fimmu.2019.01084 31178859 PMC6543837

[47] Eash KJ Means JM White DW Link DC. CXCR4 is a key regulator of neutrophil release from the bone marrow under basal and stress granulopoiesis conditions. *Blood.* (2009) 113:4711–9. 10.1182/blood-2008-09-177287 19264920 PMC2680371

[48] De Filippo K Rankin SM. CXCR4, the master regulator of neutrophil trafficking in homeostasis and disease. *Eur J Clin Invest.* (2018) 48(Suppl. 2):e12949. 10.1111/eci.12949 29734477 PMC6767022

[49] Vafadarnejad E Rizzo G Krampert L Arampatzi P Arias-Loza AP Nazzal Y Dynamics of cardiac neutrophil diversity in murine myocardial infarction. *Circ Res.* (2020) 127:e232–49. 32811295 10.1161/CIRCRESAHA.120.317200

[50] Bajpai G Bredemeyer A Li W Zaitsev K Koenig AL Lokshina I Tissue resident CCR2- and CCR2+ cardiac macrophages differentially orchestrate monocyte recruitment and fate specification following myocardial injury. *Circ Res.* (2019) 124:263–78. 10.1161/CIRCRESAHA.118.314028 30582448 PMC6626616

[51] Bajpai G Schneider C Wong N Bredemeyer A Hulsmans M Nahrendorf M The human heart contains distinct macrophage subsets with divergent origins and functions. *Nat Med* (2018) 24:1234–45. 10.1038/s41591-018-0059-x 29892064 PMC6082687

[52] Xia Y Frangogiannis NG. MCP-1/CCL2 as a therapeutic target in myocardial infarction and ischemic cardiomyopathy. *Inflamm Allergy Drug Targets.* (2007) 6:101–7. 17692033 10.2174/187152807780832265

[53] Luehmann HP Pressly ED Detering L Wang C Pierce R Woodard PK PET/CT imaging of chemokine receptor CCR5 in vascular injury model using targeted nanoparticle. *J Nucl Med.* (2014) 55:629–34. 10.2967/jnumed.113.132001 24591489 PMC4255944

[54] Hazen SL Hall CR Ford DA Gross RW. Isolation of a human myocardial cytosolic phospholipase A2 isoform. Fast atom bombardment mass spectroscopic and reverse-phase high pressure liquid chromatography identification of choline and ethanolamine glycerophospholipid substrates. *J Clin Invest.* (1993) 91:2513–22. 10.1172/JCI116487 8514863 PMC443312

[55] Li W Hsiao HM Higashikubo R Saunders BT Bharat A Goldstein DR Heart-resident CCR2(+) macrophages promote neutrophil extravasation through TLR9/MyD88/CXCL5 signaling. *JCI Insight.* (2016) 1:e87315. 10.1172/jci.insight.87315 27536731 PMC4985028

[56] Ren Y Silverstein RL Allen J Savill J. CD36 gene transfer confers capacity for phagocytosis of cells undergoing apoptosis. *J Exp Med.* (1995) 181:1857–62. 10.1084/jem.181.5.1857 7536797 PMC2192004

[57] Zlatanova I Pinto C Silvestre JS. Immune modulation of cardiac repair and regeneration: the art of mending broken hearts. *Front Cardiovasc Med.* (2016) 3:40. 10.3389/fcvm.2016.00040 27790620 PMC5063859

[58] Phillipson M Kubes P. The healing power of neutrophils. *Trends Immunol.* (2019) 40:635–47. 31160208 10.1016/j.it.2019.05.001

[59] Horckmans M Ring L Duchene J Santovito D Schloss MJ Drechsler M Neutrophils orchestrate post-myocardial infarction healing by polarizing macrophages towards a reparative phenotype. *Eur Heart J.* (2017) 38:187–97. 10.1093/eurheartj/ehw002 28158426

[60] Bharadwaj KG Hiyama Y Hu Y Huggins LA Ramakrishnan R Abumrad NA Chylomicron- and VLDL-derived lipids enter the heart through different pathways: in vivo evidence for receptor- and non-receptor-mediated fatty acid uptake. *J Biol Chem.* (2010) 285:37976–86. 10.1074/jbc.M110.174458 20852327 PMC2992231

[61] Glatz JFC Zuurbier CJ Larsen TS. Targeting metabolic pathways to treat cardiovascular diseases. *Biochim Biophys Acta Mol Basis Dis.* (2020) 1866:165879. 10.1016/j.bbadis.2020.165879 32562699

[62] Augustus A Yagyu H Haemmerle G Bensadoun A Vikramadithyan RK Park SY Cardiac-specific knock-out of lipoprotein lipase alters plasma lipoprotein triglyceride metabolism and cardiac gene expression. *J Biol Chem.* (2004) 279:25050–7. 10.1074/jbc.M401028200 15028738

[63] Augustus AS Buchanan J Park TS Hirata K Noh HL Sun J Loss of lipoprotein lipase-derived fatty acids leads to increased cardiac glucose metabolism and heart dysfunction. *J Biol Chem.* (2006) 281:8716–23. 16410253 10.1074/jbc.M509890200

[64] Li J Romestaing C Han X Li Y Hao X Wu Y Cardiolipin remodeling by ALCAT1 links oxidative stress and mitochondrial dysfunction to obesity. *Cell Metab.* (2010) 12:154–65. 20674860 10.1016/j.cmet.2010.07.003 PMC2923392

[65] Mancuso DJ Sims HF Han X Jenkins CM Guan SP Yang K Genetic ablation of calcium-independent phospholipase A2gamma leads to alterations in mitochondrial lipid metabolism and function resulting in a deficient mitochondrial bioenergetic phenotype. *J Biol Chem.* (2007) 282:34611–22. 10.1074/jbc.M707795200 17923475 PMC2980283

[66] Minkler PE Hoppel CL. Separation and characterization of cardiolipin molecular species by reverse-phase ion pair high-performance liquid chromatography-mass spectrometry. *J Lipid Res.* (2010) 51:856–65. 10.1194/jlr.D002857 19965604 PMC2842139

[67] Xu Y Malhotra A Ren M Schlame M. The enzymatic function of tafazzin. *J Biol Chem.* (2006) 281:39217–24. 17082194 10.1074/jbc.M606100200

[68] Werdich AA Baudenbacher F Dzhura I Jeyakumar LH Kannankeril PJ Fleischer S Polymorphic ventricular tachycardia and abnormal Ca2+ handling in very-long-chain acyl-CoA dehydrogenase null mice. *Am J Physiol Heart Circ Physiol.* (2007) 292:H2202–11. 10.1152/ajpheart.00382.2006 17209005

[69] Gelinas R Thompson-Legault J Bouchard B Daneault C Mansour A Gillis MA Prolonged QT interval and lipid alterations beyond beta-oxidation in very long-chain acyl-CoA dehydrogenase null mouse hearts. *Am J Physiol Heart Circ Physiol.* (2011) 301:H813–23. 10.1152/ajpheart.01275.2010 21685264 PMC3191095

[70] Pike LJ Han X Chung KN Gross RW. Lipid rafts are enriched in arachidonic acid and plasmenylethanolamine and their composition is independent of caveolin-1 expression: a quantitative electrospray ionization/mass spectrometric analysis. *Biochemistry.* (2002) 41:2075–88. 10.1021/bi0156557 11827555

[71] Khaselev N Murphy RC. Susceptibility of plasmenyl glycerophosphoethanolamine lipids containing arachidonate to oxidative degradation. *Free Radic Biol Med.* (1999) 26:275–84. 10.1016/s0891-5849(98)00211-1 9895217

[72] Skaff O Pattison DI Davies MJ. The vinyl ether linkages of plasmalogens are favored targets for myeloperoxidase-derived oxidants: a kinetic study. *Biochemistry.* (2008) 47:8237–45. 10.1021/bi800786q 18605737

[73] Dean JM Lodhi IJ. Structural and functional roles of ether lipids. *Protein Cell.* (2018) 9:196–206. 28523433 10.1007/s13238-017-0423-5 PMC5818364

[74] Thukkani AK Hsu FF Crowley JR Wysolmerski RB Albert CJ Ford DA. Reactive chlorinating species produced during neutrophil activation target tissue plasmalogens: production of the chemoattractant, 2-chlorohexadecanal. *J Biol Chem.* (2002) 277:3842–9. 10.1074/jbc.M109489200 11724792

[75] Sheedy FJ Grebe A Rayner KJ Kalantari P Ramkhelawon B Carpenter SB CD36 coordinates NLRP3 inflammasome activation by facilitating intracellular nucleation of soluble ligands into particulate ligands in sterile inflammation. *Nat Immunol.* (2013) 14:812–20. 10.1038/ni.2639 23812099 PMC3720827

[76] Batchu SN Lee SB Qadhi RS Chaudhary KR El-Sikhry H Kodela R Cardioprotective effect of a dual acting epoxyeicosatrienoic acid analogue towards ischaemia reperfusion injury. *Br J Pharmacol.* (2011) 162:897–907. 10.1111/j.1476-5381.2010.01093.x 21039415 PMC3042200

[77] Spiecker M Liao JK. Vascular protective effects of cytochrome p450 epoxygenase-derived eicosanoids. *Arch Biochem Biophys.* (2005) 433:413–20. 15581597 10.1016/j.abb.2004.10.009

[78] Huh HY Pearce SF Yesner LM Schindler JL Silverstein RL. Regulated expression of CD36 during monocyte-to-macrophage differentiation: potential role of CD36 in foam cell formation. *Blood.* (1996) 87:2020–8. 8634453

[79] Goyal T Mitra S Khaidakov M Wang X Singla S Ding Z Current concepts of the role of oxidized LDL receptors in atherosclerosis. *Curr Atheroscler Rep.* (2012) 14: 150–9. 10.1007/s11883-012-0228-1 22286193

[80] Stewart CR Stuart LM Wilkinson K van Gils JM Deng J Halle A CD36 ligands promote sterile inflammation through assembly of a Toll-like receptor 4 and 6 heterodimer. *Nat Immunol.* (2010) 11:155–61. 10.1038/ni.1836 20037584 PMC2809046

[81] Ballesteros I Cuartero MI Pradillo JM de la Parra J Perez-Ruiz A Corbi A Rosiglitazone-induced CD36 up-regulation resolves inflammation by PPARgamma and 5-LO-dependent pathways. *J Leukoc Biol.* (2014) 95:587–98. 10.1189/jlb.0613326 24338629

[82] Glinton KE Ma W Lantz CW Grigoryeva LS DeBerge M Liu X Macrophage-produced VEGFC is induced by efferocytosis to ameliorate cardiac injury and inflammation. *J Clin Invest.* (2022) 132:e140685. 10.1172/JCI140685 35271504 PMC9057589

[83] Cifarelli V Appak-Baskoy S Peche VS Kluzak A Shew T Narendran R Visceral obesity and insulin resistance associate with CD36 deletion in lymphatic endothelial cells. *Nat Commun.* (2021) 12:3350. 10.1038/s41467-021-23808-3 34099721 PMC8184948

[84] Serhan CN Chiang N Dalli J. The resolution code of acute inflammation: novel pro-resolving lipid mediators in resolution. *Semin Immunol.* (2015) 27:200–15. 10.1016/j.smim.2015.03.004 25857211 PMC4515371

[85] Love-Gregory L Abumrad NA. CD36 genetics and the metabolic complications of obesity. *Curr Opin Clin Nutr Metab Care.* (2011) 14:527–34. 21912245 10.1097/MCO.0b013e32834bbac9 PMC3295590

[86] Kintaka T Tanaka T Imai M Adachi I Narabayashi I Kitaura Y. CD36 genotype and long-chain fatty acid uptake in the heart. *Circ J.* (2002) 66:819–25. 12224819 10.1253/circj.66.819

[87] Huang CC Chang MT Leu HB Yin WH Tseng WK Wu YW Association of arachidonic acid-derived lipid mediators with subsequent onset of acute myocardial infarction in patients with coronary artery disease. *Sci Rep.* (2020) 10:8105. 10.1038/s41598-020-65014-z 32415198 PMC7229015

